# A two-step synthesis of nanosheet-covered fibers based on *α*-Fe_2_O_3_/NiO composites towards enhanced acetone sensing

**DOI:** 10.1038/s41598-018-20103-y

**Published:** 2018-01-26

**Authors:** Mahmood ul Haq, Zhen Wen, Ziyue Zhang, Shahid Khan, Zirui Lou, Zhizhen Ye, Liping Zhu

**Affiliations:** 1 State Key Laboratory of Silicon Materials, School of Materials Science and Engineering, Cyrus Tang Center for Sensor Materials and Applications, Zhejiang University, Hangzhou, 310027 China; 2 Institute of Functional Nano and Soft Materials (FUNSOM), Jiangsu Key Laboratory for Carbon-Based Functional Materials and Devices, and Joint International Research Laboratory of Carbon-Based Functional Materials and Devices, Soochow University, Suzhou, 215123 China

## Abstract

A novel hierarchical heterostructures based on *α*-Fe_2_O_3_/NiO nanosheet-covered fibers were synthesized using a simple two-step process named the electrospinning and hydrothermal techniques. A high density of *α*-Fe_2_O_3_ nanosheets were uniformly and epitaxially deposited on a NiO nanofibers. The crystallinity, morphological structure and surface composition of nanostructured based on *α*-Fe_2_O_3_/NiO composites were investigated by XRD, SEM, TEM, EDX, XPS and BET analysis. The extremely branched *α*-Fe_2_O_3_/NiO nanosheet-covered fibers delivered an extremely porous atmosphere with huge specific surface area essential for superior gas sensors. Different nanostructured based on *α*-Fe_2_O_3_/NiO composites were also explored by adjusting the volume ratio of the precursors. The as-prepared samples based on *α*-Fe_2_O_3_/NiO nanocomposite sensors display apparently enhanced sensing characteristics, including higher sensing response, quick response with recovery speed and better selectivity towards acetone gas at lower operating temperature as compared to bare NiO nanofibers. The sensing response of S-2 based α-Fe_2_O_3_/NiO nanosheet-covered fibers were 18.24 to 100 ppm acetone gas at 169 °C, which was about 6.9 times higher than that of bare NiO nanofibers. The upgraded gas sensing performance of composites based on *α*-Fe_2_O_3_/NiO nanosheet-covered fibers might be ascribed to the exclusive morphologies with large surface area, p-n heterojunctions and the synergetic performance of *α*-Fe_2_O_3_ and NiO.

## Introduction

Recently scientists have become very fascinated in finding the advanced diagnosis techniques regarding human health due to the increasingly obvious problems concerning foodstuff and the environment. Previously, researchers have proposed several testing method exploiting the examination of exhaled breath containing various inorganic and organic gases produced through the assimilation process from the human bodies^1^. Essentially, diabetes, asthma, heart sickness, kidney failure and respiratory infection are mostly associated to the concentration of exhaled acetone, nitrogen monoxide, carbon monoxide, and ammonia, respectively^2,3^. Generally, gas/liquid chromatographic analysis, cavity ring down and ion-flow tube mass spectrometry are most common and representative methods used to detect acetone in exhaled breath, which are time consuming and highly expensive techniques^4–6^. Subsequently, the developments of chemosensors for monitoring the toxic organic and inorganic gases have considered being simple and convenient method to accomplish the demand over the complex and extreme environmental system^7,8^. The resistive gas-sensor among different sorts of sensors have assumed metal oxide semiconductors (MOS) as sensing materials hold a prominent site due to their benefits; such as simplicity in processing, high sensitivity and low cost^9^.

Generally, the gas-sensitivity of p-type metal oxides was relatively lower than that of n-type metal oxides due to differences in their gas sensing mechanisms^10–12^. Presently, various effective approaches have been introduced to boost the gas-sensing features of the p-type or n-type metal oxides based sensors involving the assembly of complex and multidimensional nanostructures^13^, the materialization of nanocomposites^14^, the loading of noble metal catalysts^15^ and the aliovalent ions doping^16^. P-type nickel oxide (NiO) with 3.4 eV energy gap and n-type hematite (*α*-Fe_2_O_3_) with 2.0 eV energy gap are dissimilar imperative functional materials existing as various morphological structures^17–20^, which have received general attention because of their unique physical and chemical features leading to their great performance in wide range of applications including dye-sensitized solar cells^21,22^, catalysis^23,24^, gas sensors^25,26^, electrodes^27,28^, magnetic materials^29,30^ and electrochemical supercapacitors^31,32^ *etc*.

The use of a single metal oxide can determine a low gas-sensitivity because of insufficient exposing area and low electron transmission due to surface features^33^. One of the best significant ways to combine the different physical and chemical features of individual components into one scheme is the fabrication of nanocomposites based on the combination of p-type and n-type metal oxides^34,35^. As regards gas sensing applications, the superior functional performances of the nanocomposites as compare to single-phase metal oxides are mostly attributed to the creation of an inner electric field at the p/n junction interface^36–38^. A number of approaches had been established to prepare NiO and α-Fe_2_O_3_ based nanomaterials including the hydrothermal method^39,40^, the pulsed laser deposition method^41^, the solution plasma method^42^, the micro emulsion method^43^, chemical vapor deposition, the template-assisted approach^44^ and electrospinning^45^.

Herein, we report a novel two-step method for the fabrication of nanocomposites based on α-Fe_2_O_3_/NiO nanosheet-covered fibers using electrospinning techniques with the support of hydrothermal method in order to compare and evaluate the structural properties on the gas detecting features towards toxic gases, such as acetone, ethanol, methanol, xylene, toluene and benzene. The crystallinity, morphological structure, chemical composition and gas sensing features of the composites based on *α*-Fe_2_O_3_/NiO nanosheet-covered fibers were discussed.

## Results and Discussion

**Figure 1:**
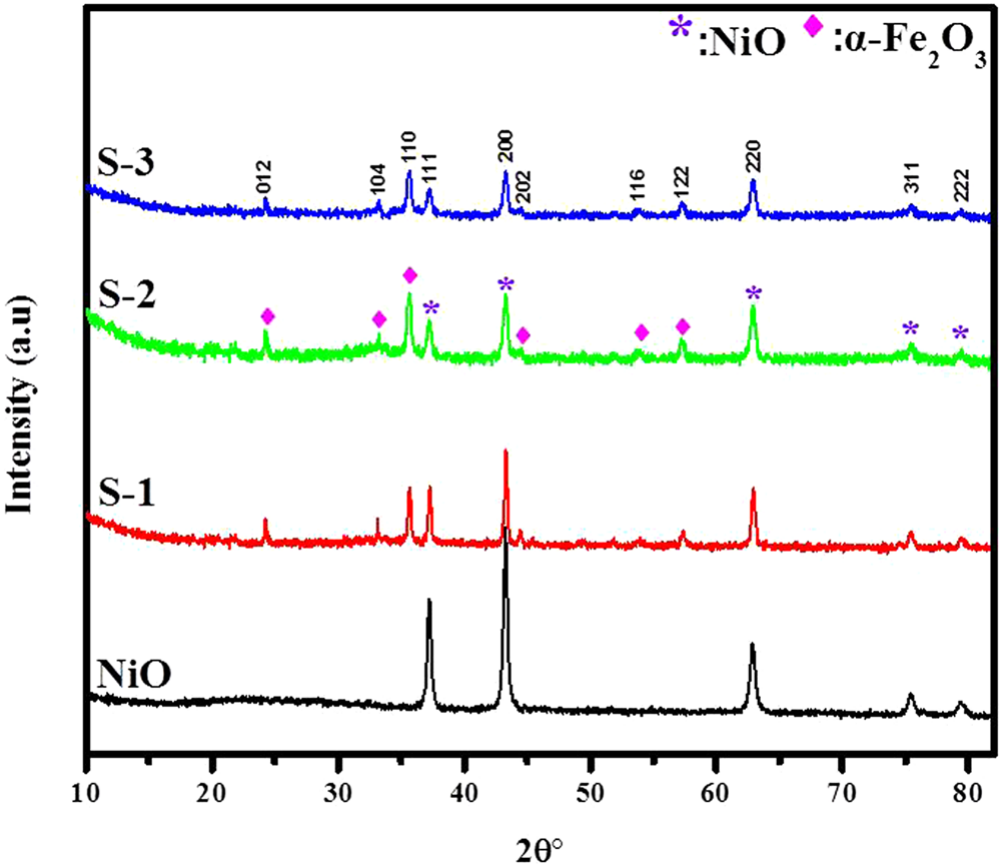
XRD pattern of pure NiO nanofibers, composites S-1, S-2 and S-3 based on *α*-Fe_2_O_3_/NiO nanosheet-covered fibers with different Fe concentrations.

Crystal, morphological structure and compositional features of the as-prepared bare NiO and the nanocomposites based on α-Fe_2_O_3_/NiO samples were described by using XRD, SEM, TEM, EDS, XPS and BET examination. The composite NiO-PVP nanofibers were turned into pure NiO nanofibers after annealing at 600 °C in air. The XRD pattern of the NiO nanofibers display face-centered cubic phase geometry, which matched well with the standard card NiO (JCPDS Card No. 47-1049), as shown in Fig. 1. We implemented a hydrothermal method to grow α-Fe_2_O_3_ nanosheets on the surface of electrospum NiO nanofibers and the diffraction pattern with phase purity of as-produced composites based on α-Fe_2_O_3_/NiO nanosheet-covered fibers were presented in Fig. 1. All the XRD peaks present in the composites based on α-Fe_2_O_3_/NiO corresponds well with the standard crystallographic patterns of the face-centered cubic phase of NiO (JCPDS Card No. 47-1049) and the rhombohedral phase of α-Fe_2_O_3_ (JCPDS Card No. 33-0664) without any extra peaks, which indicates the high purity of α-Fe_2_O_3_/NiO heterostructures. With the increasing of Fe contents, the intensity of diffraction peaks decreases and became broader with a clear phase separation in sample S-2 and S-3 as compare to S-1 based α-Fe_2_O_3_/NiO nanocomposites, which results in reduction of the crystallite size as shown in Table 1. These facts demonstrate that ions can be isolated at the junction and the microstrain established in the composites nanosheet-covered fibers increases while the particle size decreases^46^.

**Table 1:** Compositional and structural parameter of as-prepared samples based on α-Fe_2_O_3_/NiO nanocomposites.

Sample Code.	Atomic Percent % (Fe^+3^)	Morphology	Crystalline Parameter hkl & D (nm)	Surface Area (m^2^g^−1^)
NiO	/	Nanofibers	NiO: (200) = 24.66 nm	30
S-l (α-Fe_2_O_3_/NiO)	2.6 at.%	Nanosheet-covered fibers	*α*-Fe_2_O_3_: (110) = 20.86 nm NiO:(200) = 23.44 nm	41
S-2 (α-Fe_2_O_3_/NiO)	4.4 at.%	Nanosheet-covered fibers	*α*-Fe_2_O_3_: (110) = 18.98 nm NiO:(200) = 21.56 nm	47
S-3 (α-Fe_2_O_3_/NiO)	8.9 at.%	Nanosheet-covered fibers	*α*-Fe_2_O_3_: (110) = 18.75 nm NiO:(200) = 21.45 nm	49

**Figure 2:**
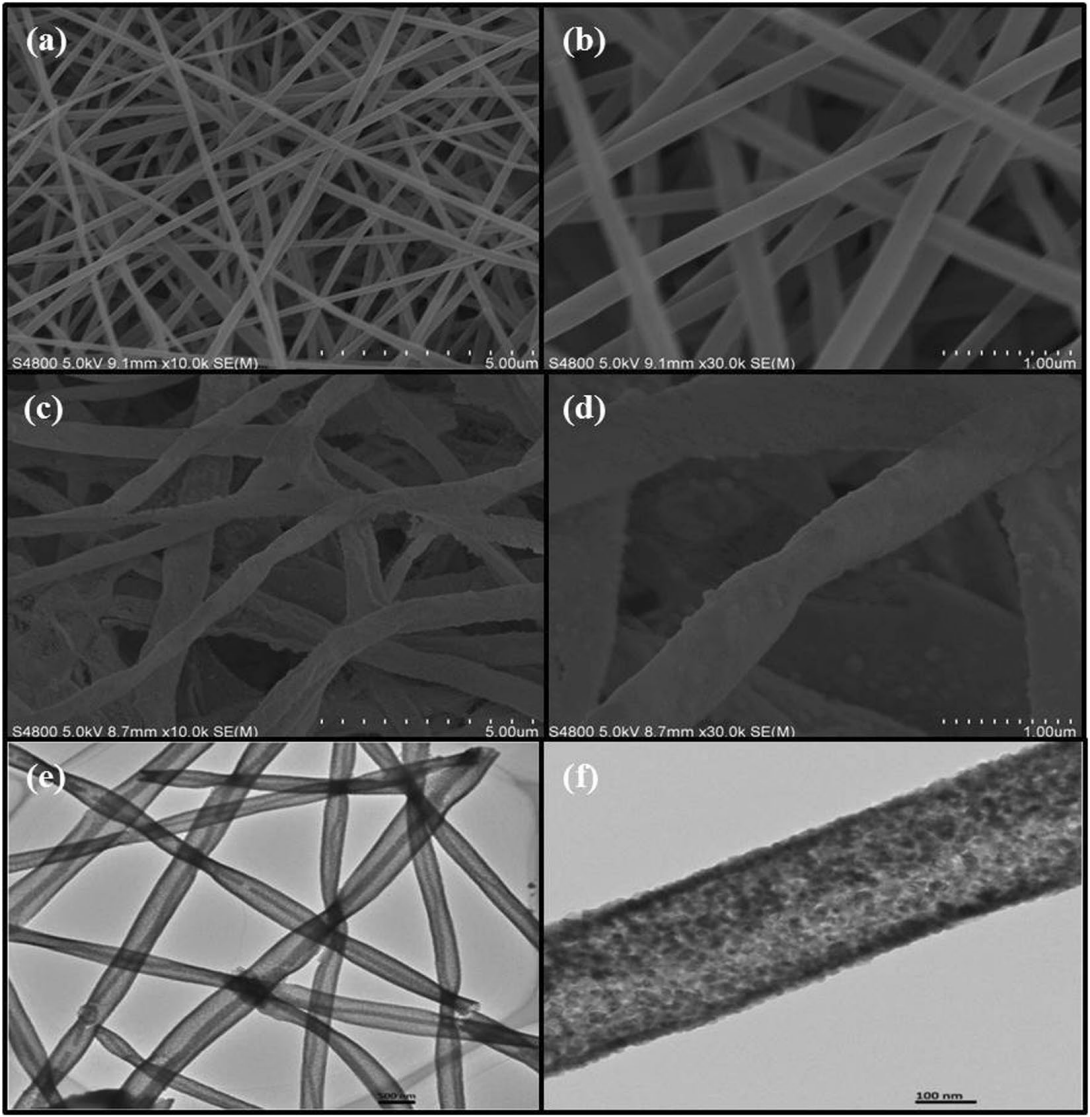
Typical SEM images of pure NiO nanofibers **(a,b)** Before calcination, **(c,d)** After calcination and **(e,f)** TEM images of pure NiO nanofibers.

The SEM images of the pure NiO sample before and after calcination comprised of ultralong nanofibers with uniform diameter having a smooth surface shown in Fig. 2a–d. The spreading of the nanofibers were enough random with no clear pattern. The average length and diameter range of NiO nanofibers were about ~13–15μm long and ~0.5–0.6 μm thick. Moreover, the transmission electron microscopy images of NiO nanofibers specifies that the NiO nanofibers were composed of clear smooth surfaces shown in Fig. 2e and f. The nanofibers were ultralong and continuous with random alignment.

**Figure 3:**
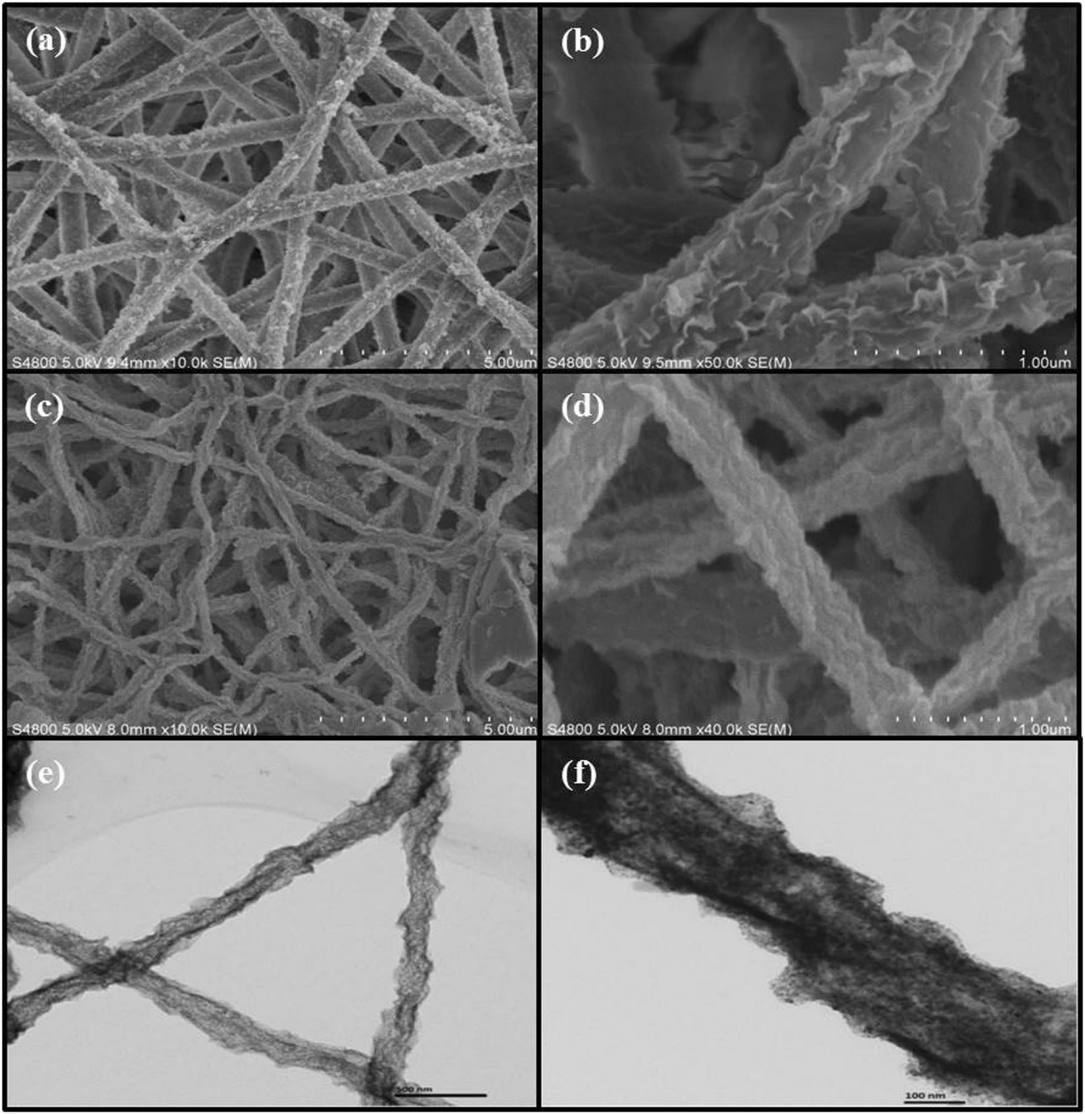
SEM images of composites S-2 based on α-Fe_2_O_3_/NiO nanosheet-covered fibers **(a,b)** Before calcination, **(c,d)** After calcination and **(e,f)** TEM images of composites S-2 based on *α*-Fe_2_O_3_/NiO nanosheet-covered fibers.

Typically, a single sample S-2 based α-Fe_2_O_3_/NiO composites were chosen for examination under scanning electronic microscope. Figure 3a–d display the SEM images with low and high-magnification of the sample S-2 based nanocomposites before and after calcination, which shows that the electrospum ultralong nanofibers architectures of NiO were maintained but, only growth of α-Fe_2_O_3_ nanosheets as branches over the stem of NiO nanofibers after the hydrothermal treatment. The average length and diameter of S-2 samples were ~13 μm and a diameter of ~0.4 μm thick. The α-Fe_2_O_3_ nanosheets grew outwardly from the surfaces of NiO nanofibers to form the branch heterostructure. The typical SEM images of the samples S-1 and S-3 based α-Fe_2_O_3_/ NiO nanocomposites were shown in Fig. S1. The electrospum nanosheet-covered fibers were uniform in diameter having a rough surface with random alignment. Further investigation of morphology of the sample S-2 based nanocomposites was made by TEM, a similar distinct nanosheet-covered fibers structure could be clearly identified as shown in Fig. 3e and f, which clearly matches with SEM images. It can be realized that the nanosheet-covered fibers have a coarser morphology and are composed of inter-associated grains. All the samples based on α-Fe_2_O_3_/NiO nanocomposites, the nanofibers were too long, continuous and random pattern with readily growth of α-Fe_2_O_3_ nanosheet as branches. The increase of Fe content leads to the amendments in the nanosheet-covered fibers surface with the creation of porous hollow structure among the inter-related nanoparticles. These porous structures in the nanocomposites were also related to the putrefaction of organic phase^47^. Thus, these porous morphologies leads to the improved specific surface area and it benefit towards superior sensing features of the sensors.

The elemental composition of bare NiO nanofibers and α-Fe_2_O_3_/NiO nanosheet-covered fibers with different Fe concentrations was performed using EDX’s analysis was shown in Fig. S2. All the spectra of as-synthesized samples designate the presence of Ni, Fe and O elements in the samples. The atomic percentages of Fe in α-Fe_2_O_3_/NiO nanosheet-covered fibers were 2.6 at% (S-1), 4.4 at% (S-2) and 8.9 at% (S-3), respectively. The rise of oxygen content in composites samples with Fe amounts exposed the existence of oxide phase like α-Fe_2_O_3_ as observed in XRD pattern. Obviously, the stem was NiO nanofibers and the outgrowth branches were α-Fe_2_O_3_ nanosheets according to the spatial spreading of Ni and Fe signals in the EDS spectrum.

**Figure 4:**
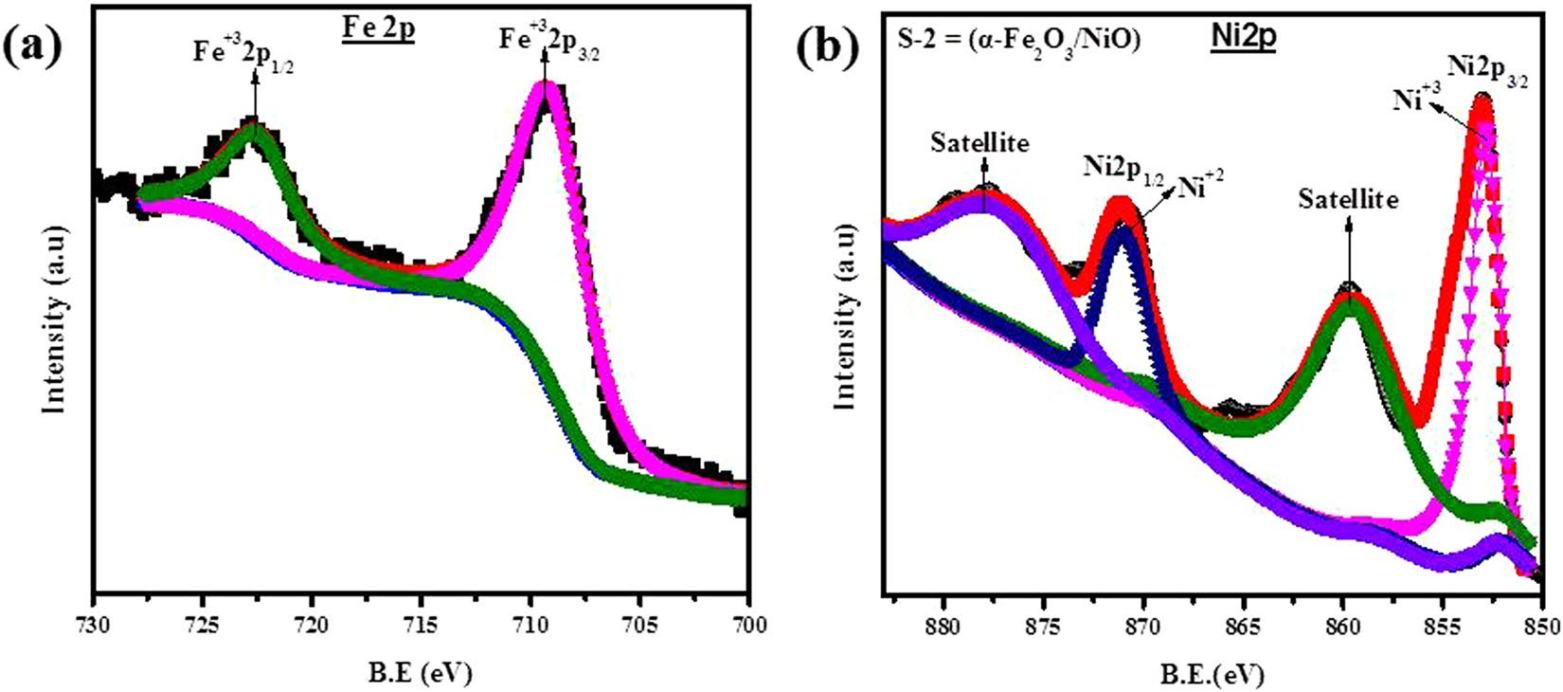
XPS spectra for composites S-2 based on α-Fe_2_O_3_/NiO nanosheet-covered fibers **(a)** Fe-2p_3/2_ and **(b)** Ni-2p_3/2_.

To further characterize the composition of the S-2 based α-Fe_2_O_3_/NiO composites, X-ray photoelectron spectroscopy (XPS) was evaluated. It can be realized from the XPS wide spectrum that the S-2 based nanocomposites contained Ni, Fe, O and C elements as shown in Fig. S3. The C signal could be ascribed to adventitious hydrocarbon. The complete XPS spectrums of the Fe-2p range and the Ni-2p range were presented in Fig. 4a and b. In case of Fe-2p spectrum, the Fe-2p peak could be distributed into two signals as presented in Fig. 4a. Fe-2p_3/2_ and Fe-2p_1/2_ signals were focused at 709 eV and 722.6 eV, respectively, which agreed with the electronic state of α-Fe_2_O_3_^48^. In case of Ni-2p spectrum, the Ni-2p peak could be distributed into four signals as presented in Fig. 4b. The binding energy peaks at 852 eV and 859 eV were ascribed to the Ni-2p_3/2_ and its satellite peak while, the peaks at 870 eV and 877 eV were ascribed to Ni-2p_1/2_ and its satellite. The Ni-2p_3/2_ peaks were credited to Ni^3+^ while, Ni-2p1/2 peaks were ascribed to Ni^2+^ ^49^. The XPS results further specified that the ultimate products were composed of α-Fe_2_O_3_ and NiO.

**Figure 5:**
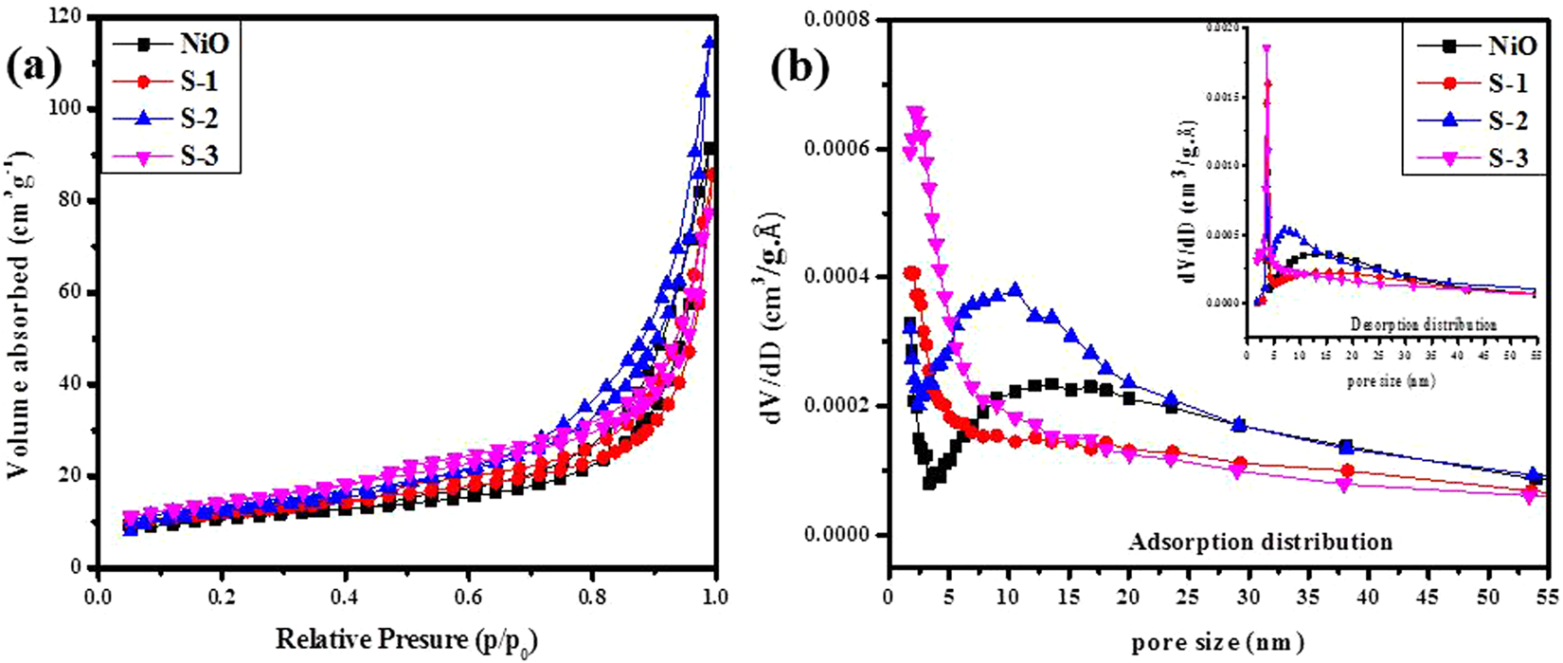
(**a**) Nitrogen adsorption-desorption isotherms and **(b)** The corresponding adsorption-desorption pore-size distribution curves of Pure NiO, S-1, S-2 and S-3 based *α*-Fe_2_O_3_/NiO composites.

Likewise, the BET with N_2_ adsorption-desorption analysis were explored for further examination of specific surface area and pore size distribution of the bare NiO nanofibers and the sample S-1, S-2 and S-3 based α-Fe_2_O_3_/NiO nanosheet-covered fibers. Figure 5a presents the N_2_ adsorption-desorption isotherms of all prepared samples, which expose a type IV adsorption isotherm with a H3-type hysteresis loop at different relative pressure ranges. The consistent specific surface areas of bare NiO nanofibers and the sample S-1, S-2 and S-3 based α-Fe_2_O_3_/NiO nanosheet-covered fibers were 30 m^2^g^−1^; 41 m^2^g^−1^; 47 m^2^g^−1^and 49 m^2^g^−1^, respectively. From Fig. 5b, the adsorption pore size distribution curve of all prepared samples indicates the mesopores with broad size distribution, which clearly match with adsorption-desorption isotherms (Fig. 5a). The desorption distribution curve inset of Fig. 5b shows sharp pore size distribution which might be false peaks. The spreading of the pore diameter of S-2 based α-Fe_2_O_3_/NiO nanocomposites are more concentrated with a large number of mesopores (peak pore at ca. 2~10 nm) and a small number of large mesopores and macropores. The great improvements in specific surface area of nanocomposites based on α-Fe_2_O_3_/NiO samples may arise from the voids among close-packing nanosheet-covered fibers and mesoporous voids between the nano-crystallites^50^.

### Gas-sensing properties

We fabricated gas sensors based on bare NiO nanofibers and the sample S-1, S-2 and S-3 based α-Fe_2_O_3_/NiO nanosheet-covered fibers, in order to study the gas sensing properties towards various toxic gases. The transient sensing response characteristics of as-synthesized sensors towards acetone, ethanol, methanol, xylene, toluene and benzene were investigated. The electric resistance of the as-prepared sensors increased sharply on the injection of target gas and then decreased promptly and recovered to its original value after the target gas was released, which displays the sensing performance of p/n type semiconducting sensors.

**Figure 6:**
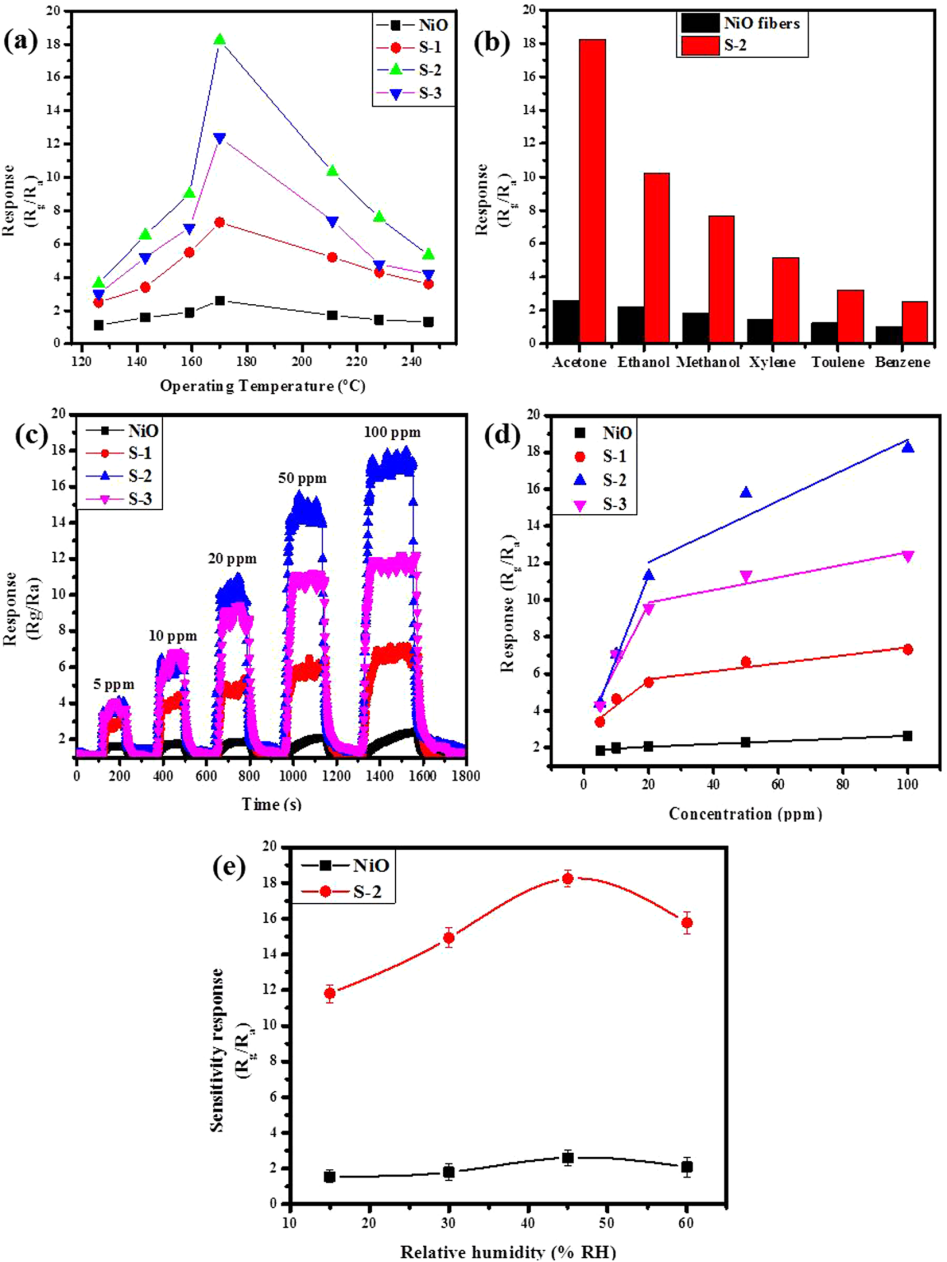
(**a**) The transient gas-responses of the sensors based on pure NiO, composites S-1, S-2 and S-3 based on α-Fe_2_O_3_/NiO nanosheet-covered fibers vs operating temperatures to 100 ppm acetone. **(b)** Gas responses of pure NiO and composites S-2 based on α-Fe_2_O_3_/NiO sensors to 100 ppm various target gases (Acetone; Ethanol; Methanol; Xylene, Toluene and Benzene) at 169 °C. **(c)** Real-time sensing-response curves of the sensors based on pure NiO, composites S-1, S-2 and S-3 based on α-Fe_2_O_3_/NiO nanosheet-covered fibers to various acetone concentrations at 169 °C. **(d)** Gas-responses of four sensors as a function of the acetone concentrations at 169 °C. Solid lines show the linear polynomial fitting of the experimental data. **(e)** The change of the sensitivity response of sensors based on pure NiO and S-2 based nanocomposites in different test chamber relative humidity (Error bars represent the standard error obtained in three measurements).

First of all, the maximum transient gas-sensing responses of the sensors based on bare NiO nanofibers and the sample S-1, S-2 and S-3 based α-Fe_2_O_3_/NiO nanosheet-covered fibers towards acetone gas were explored at diverse functioning temperatures from 125 to 250 °C to examine the optimal Fe doping amount into α-Fe_2_O_3_/NiO nanocomposites as well as the correlation between gas response and functioning temperature, as shown in Fig. 6a. Apparently, the volcano-shaped connection between gas responses and functioning temperature was perceived for all the samples, and the best functioning temperature of each sample was 169 °C. Meanwhile, the gas response was greatly improved due to increasing Fe concentration into α-Fe_2_O_3_/NiO nanocomposites. The sensing responses of the sensors based on bare NiO nanofibers and the sample S-1, S-2 and S-3 based α-Fe_2_O_3_/NiO nanosheet-covered fibers to 100 ppm acetone at 169 °C were 2.64, 7.32, 18.24 and 12.41, respectively. The result exposed that the sensor based on S-2 based α-Fe_2_O_3_/NiO nanosheet-covered fibers presented the maximum response to 100 ppm acetone and the value was about 6.9 times superior to that of bare NiO nanofibers.

Subsequently, the gas responses of sensors based on bare NiO nanofibers and the sample S-2 based α-Fe_2_O_3_/NiO nanosheet-covered fibers to 100 ppm of various target gases at 169 °C were examined. The target gases comprised acetone, ethanol, methanol, xylene, toluene and benzene. As shown in Fig. 6b, the sensor S-2 based α-Fe_2_O_3_/NiO nanosheet-covered fibers exposed superior response for all tested gases compared to that of bare NiO nanofibers. Moreover, the response of both sensors to acetone was obviously better than that to other gases. The sensing selectivity of obtained sensor S-2 based α-Fe_2_O_3_/NiO nanocomposites for acetone detection can be explained by the relatively low bond dissociation energy of acetone molecules (393 kj/mol) as compare to other volatile organic compounds (VOCs) gases^51^. Meanwhile, large number of oxygen contents (O_C_ and O_V_) and the formation of p-n junction on the surface of the Fe_2_O_3_/NiO nanocomposites make it release more electrons during redox reaction between acetone molecules and chemically adsorbed oxygen ions, which reflects its selective detection.

The real-time sensing response of as-synthesized gas sensors based on bare NiO nanofibers and the sample S-1, S-2 and S-3 based α-Fe_2_O_3_/NiO nanosheet-covered fibers at 169 °C towards different acetone concentration ranges from 5–100 ppm shown in Fig. 6c. As all the sensors based on sample S-1, S-2 and S-3 based α-Fe_2_O_3_/NiO nanosheet-covered fibers displayed enhanced sensing response for acetone gas compared to the bare NiO nanofibers. It has been perceived that the response increased with increasing acetone concentration from 5 to 100 ppm for all the prepared sensors and the growth gradually slowed down with increasing acetone than 100 ppm. Among the whole fabricated series, the response of the sensor based on the sample S-2 α-Fe_2_O_3_/NiO nanosheet-covered fibers were apparently higher than that of the other samples to various acetone concentrations we tested, shown in the Fig. 6d. Moreover, we could find that the response of the sensor S-2 based on α-Fe_2_O_3_/NiO nanocomposites did not tend to saturation gradually when the acetone concentration was raised to 100 ppm, although the increasing trend slowed down with the further increase of the acetone concentration. The result exposed that all the sensors presented tremendous response and recovery features with respect to diverse acetone concentrations ranging from 5 to 100 ppm. The response and recovery features of all the synthesized sensors to 100 ppm acetone at 169 °C were presented in Fig. S4. Consequently, the sensor S-2 based α-Fe_2_O_3_/NiO composites have displayed quick response and recovery times of 26 s and 37 s, respectively, to 100 ppm acetone at 169 °C. The relatively rapid response and recovery of S-2 based nanocomposites contribute to the real-time detection of acetone gas.

The acetone sensitivity of sensor based on NiO nanofibers and S-2 based α-Fe_2_O_3_/NiO nanosheet-covered fibers were explored in an operating temperature of 169 °C for various known value relative humidity 15, 30, 45 and 60% HR, as plotted in Fig. 6e. According to the two direct interaction mechanisms proposed by Heiland and Kohl^52^, water vapour provides the essential conditions for oxygen adsorption, electrons and oxygen vacancies. At a certain extent, the water adsorption could accelerate the oxygen adsorption. The variation in the total concentration of adsorption sites [S_t_] is shown as:

1 $$[{S}_{t}]={[S}_{{\rm{t0}}}]+{{\rm{k}}}_{{\rm{0}}}.{\rho }_{{H}_{2}O}$$

where [S_t0_] is intrinsic concentration of adsorption sites, k_0_ is adsorption constant for water vapour and ${\rho }_{{H}_{2}O}$ is the partial pressure of water vapour, respectively. The partial pressure of water vapour (${\rho }_{{H}_{2}O}$) is proportional to the mass of relative humidity (RH). With the rises of relative humidity, the total concentration of adsorption sites [S_t_] increases, and the rate of coverage of hydroxyl groups and oxygen species altered. Thus, the relative humidity sensitivity of sensors based on NiO and S-2 based composites depends on the relative surface distribution, coverage of hydroxyl groups and oxygen species^53^. When the relative humidity is 15 and 30% HR, the sensitivity of both NiO and S-2 sensor is low, due to less number of hydroxyl groups and oxygen species on the surface of sensing materials. The best sensitivity response to acetone is in 45%, which is contributed to the small coverage of hydroxyl groups that cannot constrain the acetone adsorption and the coverage of oxygen species increase. When the relative humidity increases up to 60% RH, the sensitivity response starts to decrease, which might be due to large coverage of hydroxyl groups and the adsorption of oxygen can be limited. The superior humidity sensing response of sensors based on S-2 to that of NiO might be due to the impact of porosity that boosted the diffusion of water vapors.

In order to reflect the enhanced sensing performance of as-synthesized sensors, the results obtained in this study were quantitatively compared with those stated by many research groups about n-type *α*-Fe_2_O_3_ and p-type NiO based sensors towards acetone, listed in Table 2. The gas response of S-2 based α-Fe_2_O_3_/NiO nanosheet-covered fibers was the highest at low acetone concentration and relatively lower operating temperature of 169 °C, showing comparatively higher efficiency than those reported in the given literature^16,54–56^. Therefore, it is believed that novel S-2 based *α*-Fe_2_O_3_/NiO nanosheet-covered fibers prepared in this study provide great interest for the further analysis in the field of gas-sensing application.

**Table 2:** Comparison of the sensing performance between the current works with previously reported results^16,54–56^.

Materials	Acetone Conc. (ppm)	Operating temperature (°C)	Sensing response (R_g_/R_a_)	Ref.
Fe-Doped Ordered Mesoporous NiO	50	240	3.9	^16^
Flower-like NiO-decorated ZnO microstructures	100	300	23.5	^54^
p-NiO/n-ZnO heterostructure	100	330	12	^55^
rGO/-Fe_2_O_3_composites	100	225	13.9	^56^
S-2 based α-Fe_2_O_3_/NiO nanosheet covered fibers	100	169	18.34	This work

### Gas-sensing mechanism

**Figure 7:**
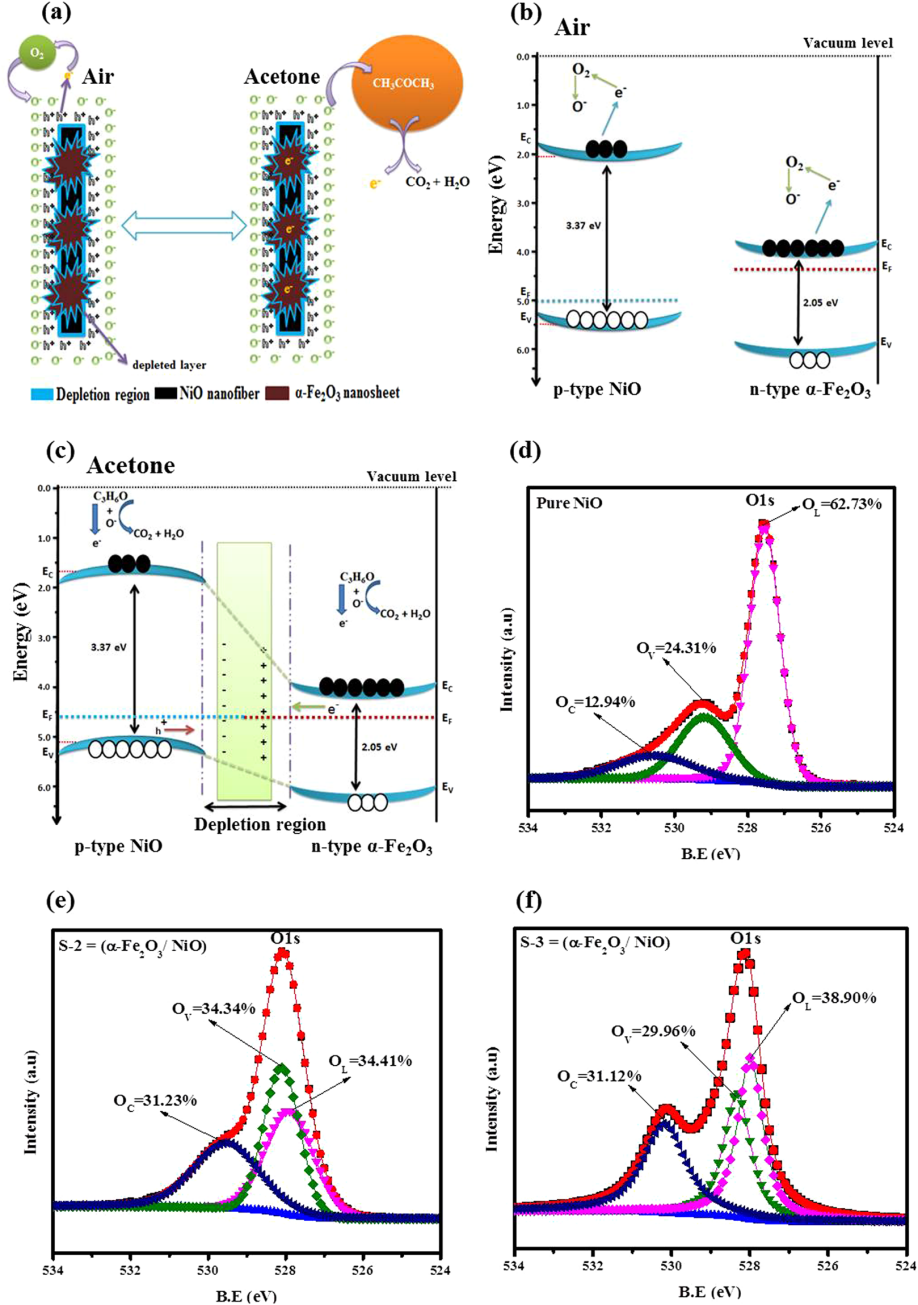
(**a**) Schematic diagrams of the acetone sensing mechanisms of heterostructure α-Fe_2_O_3_/NiO nanosheet-covered fibers in the presence of air and target acetone gas. The proposed energy band structure; (**b**) NiO and α-Fe_2_O_3_ in air before combination and (**c**) α-Fe_2_O_3_/NiO heterojunction in acetone. XPS spectra for O 1 s; **(d)** Bare NiO nanofibers, **(e)** S-2 based and **(f)** S-3 based on α-Fe_2_O_3_/NiO nanosheet-covered fibers.

In order to better understanding of the enhanced gas sensing features of sensors based on α-Fe_2_O_3_/NiO nanosheet-covered fibers, the gas sensing mechanism of SMOs is presented first. The simple sensing mechanism of n-type or p-type SMOs broadly involves the variation in electrical conductivity/resistivity due to the chemical reaction of gas molecules with the surface involving gas adsorption, surface reaction, and desorption processes, which can be well implicit by the depletion layer or space-charge model^57,58^. Generally, the adsorption and desorption of target gas molecules on the surface of SMO-based sensing materials can lead to the reaction process of electron exchanges, which are transmitted by the surface adsorbed oxygen species O^δ−^ (O_2_^−^, O^−^ and O^2−^)^59,60^. Such electron shifting causes variations in the resistance of sensing devices by making changes in the thickness of their depletion layers. Thus, the sensing properties can be upgraded by enhancing its resistance deviation. The graphic illustration of the acetone gas sensing mechanism on the surface of sensors based on heterostructure α-Fe_2_O_3_/NiO nanosheet-covered fibers in the presence of air and target gas was illustrated in Fig. 7a.

The enhanced sensing properties of sensors based on heterostructure α-Fe_2_O_3_/NiO nanosheet-covered fibers as compare to bare NiO fibers may be attributed to the creation of p-n junction between p-type NiO nanofibers and n-type α-Fe_2_O_3_ nanosheets. First, we used (α)^2^(hʋ)^2^~hʋ relation curve to compute the energy band gap of NiO nanofibers and α-Fe_2_O_3_ nanosheet from their UV-vis spectrum, as shown in Fig. S5. The band gaps calculated for NiO and α-Fe_2_O_3_ were 3.37 and 2.05 eV, respectively. Then we mentioned some literatures to get the Fermi level (NiO: 5.0 eV, α-Fe_2_O_3_: 4.39 eV), valence band of NiO (5.5 eV) and conduction band of α-Fe_2_O_3_ (4.09 eV)^61–65^. According to this data, the energy band configuration of NiO and α-Fe_2_O_3_ in air before combination had been drawn and presented graphically in Fig. 7b. After the formation of heterojunction, the energy band configuration of the composites based on *α*-Fe_2_O_3_/NiO in acetone had been drawn and presented graphically in Fig. 7c. A relative opposite motion of charge carriers took place at the junction of p-type NiO and n-type α-Fe_2_O_3_ to obtain equalization of Fermi levels, which results in band bending. As a consequence of generation of an electric field in the space charge region nearby the p-n interfaces, the energy bands in the side of NiO bend downwards and the energy bands in the side of α-Fe_2_O_3_ bend upwards. Instantaneously, an hole accumulation layer had been formed on the side of α-Fe_2_O_3_ while electronic depletion layer formed on the side of NiO. The oxygen molecules from air adsorbed on the surface of sensors based on α-Fe_2_O_3_/NiO nanocomposites will capture electrons from the conduction bands of both α-Fe_2_O_3_ and NiO and produce oxygen ions O^δ−^ adsorbed on the surface, which results in the further widening of electron depletion layer and hole depletion layer on the surface of α-Fe_2_O_3_ and NiO metal oxides, respectively. Upon exposure to acetone gas, the acetone molecules react with chemically adsorbed oxygen ions O^δ−^and breakdown into CO_2_ and H_2_O with release of the captured electrons back to Ni vacancies, thus result in the upsurge of the resistance of the composites α-Fe_2_O_3_/NiO nanosheet-covered fibers. Consequently, the sensitivity response of the S-1, S-2 and S-3 based α-Fe_2_O_3_/NiO nanosheet-covered fibers compared to bare NiO nanofibers toward acetone test gas clearly amplified due to the p-n heterojunction effect which results in the increment of the initial resistance and the degeneration in the equivalent hole concentration of the α-Fe_2_O_3_/NiO nanocomposites.

As the differences in sensing properties among samples is associated to the surface chemical composition and microstructure of the as-prepared sensors. Consequently, the O 1 s peaks of bare NiO, S-2 and S-3 based α-Fe_2_O_3_/NiO nanosheet-covered fibers were asymmetrically fitted into three dissimilar components^66^, presented in Fig. 7d–f. The binding energies at about 527.55 eV (O_L_), 529.24 eV (O_V_), and 530.60 eV (O_C_) with ±0.2 eV errors, respectively, were ascribed to lattice oxygen (O_L_), oxygen deficient regions (O_V_), and chemisorbed oxygen species (O_C_). The relative percentages of O_L_, O_V_, and O_C_ components were approximately 62.73%, 24.31% and 12.94% in the bare NiO nanofibers, 34.41%, 34.34% and 31.23% in the S-2 based α-Fe_2_O_3_/NiO nanocomposite, while they were 38.90%, 29.96% and 31.12% in the S-3 based α-Fe_2_O_3_/NiO nanocomposite. As the sensor based on S-2 composite has large number of oxygen contents such as huge number of chemisorbed oxygen (O_C_) and vacant oxygen (O_V_) on its surface, which provides comparatively more active sites and stronger adsorption capability for acetone gas to release more electrons during redox reaction as compare to bare NiO and S-3 based sensor, which suggests qualitatively that S-2 based α-Fe_2_O_3_/NiO nanocomposites display an optimal sensing behavior with fast response-recovery times^16,66^. Thus, the enhanced gas-sensing behavior of sensors based on α-Fe_2_O_3_/NiO nanocomposites might be attributed due to the small size effect of α-Fe_2_O_3_ nanosheets, large surface morphology with numerous oxygen components and the synergetic effect prompted by the compact interfacial interaction between α-Fe_2_O_3_ and NiO heterojunctions.

## Conclusion

The ultralong NiO nanofibers were synthesized by a facile electrospum technique and then further functionalized by decorating α-Fe_2_O_3_ nanosheets using an easy hydrothermal strategy. The electron microscopic images exposed that the α-Fe_2_O_3_ nanosheets were deposited epitaxially on the surfaces of NiO nanofibers to produce α-Fe_2_O_3_/NiO nanosheet-covered fibers. The gas sensors were fabricated from the as-produced samples based on α-Fe_2_O_3_/NiO nanocomposites, which exhibited enhanced sensing response and excellent selectivity toward acetone gas at relatively low temperature compared to bare NiO nanofibers. The sensing response of S-2 based α-Fe_2_O_3_/NiO nanosheet-covered fibers were 18.24 to 100 ppm acetone gas at 169 °C, which was about 6.9 times higher than that of bare NiO nanofibers. The upgraded gas sensing performance of nanocomposites based on α-Fe_2_O_3_/NiO nanosheet-covered fibers could be attributed to the unique large surface morphology, p-n heterojunctions and the synergetic performance of α-Fe_2_O_3_ and NiO.

## Methods

### Materials

Nickel (||) chloride hexahydrate, NiCl_2_•6H_2_O (98.0%); Iron (|||) chloride hexahydrate, FeCl_3_•6H_2_O (98.5%); Anhydrous Sodium carbonate, Na_2_CO_3_ (99.8%); Polyvinyl pyridine, PVP (Average molecular weight 1300, 000); Dimethylformamide, DMF; Absolute ethanol and Acetone (99.0%) were bought from Sigma Aldrich Company.

### Preparation of the electrospum NiO nanofibers

The pure NiO nanofibers were synthesized by electrospinning procedure followed by annealing. In this synthesis, 1 g NiCl_2_•6H_2_O was mixed with 4 g DMF and 6 g ethanol under stirring for 20 minutes. Afterward, 1.5 g of PVP was added into the above solution under vigorous stirring for 4 hour. The precursor’s solution was placed statically for one day to eliminate the gas bubbles before electrospinning. Then, the precursors solution was loaded into a glass syringe with a stainless steel needle connected to a diverse high voltage power range upto 30 kV. A smooth steel plate covered with aluminum foil was used as collector to collect the fibers. A 13 kV was provided between anode (needle) and cathode (collector) at a distance of 20 cm with feeding rate of 0.06 mm/min. Finally, the as-spum PVP-NiO nanofibers were calcined at 600 °C for 3 hours in air in order to eliminate PVP to get pure NiO nanofibers.

### Hydrothermal growth of α-Fe_2_O_3_ nanosheet on the NiO fibers

The composites based on α-Fe_2_O_3_/NiO nanosheet-covered fibers were synthesized by hydrothermal procedure followed by calcination. A 0.1 g of FeCl_3_•6H_2_O and 0.5 g anhydrous Na_2_CO_3_ were dissolved in 20 ml deionized water under stirring condition for 20 minutes. A suitable amount of 0.6 g NiO fibers mat was mixed to the above FeCl_3_ solution. Then, the solution was shifted into a 25 ml Teflon-based autoclave, wrapped and incubated at 80 °C for 4 h. Finally, the autoclave was refrigerated to room temperature and the fiber products were collected by washing with distilled water and ethanol to eliminate the unwanted soluble ions. Finally, the obtained products dried out at 70 °C in vacuum and then calcined in a muffle furnace at 500 °C through a heating rate of 2.0 °C/min and further annealed at 500 °C for 2 hour. Subsequently, a suitable amounts of FeCl_3_.6 H_2_O (0.3 g and 0.6 g) were used in synthesis along with other additives mentioned earlier for comparative studies. The concentration of Fe content in these three as-prepared nanocomposites based on α-Fe_2_O_3_/NiO materials is summarized in Table 1.

### Characterization

The as-produced bare NiO and nanocomposites based on α-Fe_2_O_3_/NiO samples were examined using various characterizing techniques. The X-ray diffraction patterns (XRD) of all samples were collected by using the Empyrean 200895 model pro-equipped with copper Kα radiation source in the 2θ range of 10–80°. The scanning electron microscope (SEM) with model Hitachi S-4800 attached to Energy Dispersive X-rays (EDX) spectroscopic system as well as transmission electron microscope (TEM) with model JEOL, JEM-1010 were operated to observe the size and structural morphologies of all the prepared samples. The doping concentration of Fe in various composites based on α-Fe_2_O_3_/NiO samples were studied by energy dispersive x-ray spectroscopy (EDX’s).The X-ray photoelectron spectroscopy (XPS) characterization of the samples were achieved with Mg Kα X-ray source using an analytical AXIS SUPRA model. The specific surface area investigation of all the samples were achieved by Brunauer–Emmett–Teller method (BET) with N_2_ gas adsorption through an Autosorb-IQ2-MP surface analytical instrument.

### Gas-sensors fabrication and measurement

The comprehensive of gas-sensors fabrication and measurement can be found in our earlier report^67^. The gas sensors were made-up of the thick films obtained from the powder suspension of the as-produced bare NiO and nanocomposites based on α-Fe_2_O_3_/NiO samples. Each sample was mixed in separate beaker containing ethanol and ultra-sonicated into slurry, and then it was pasted onto an Al_2_O_3_ ceramic tube by using brush to form a thick film between Au electrodes on the alumina ceramic tube. The thickness of prepared films is about 20–30 μm with the diameter of the tube is about 1.2 mm and the space between two parallel electrodes is ∼6 mm. In our case, the gas-sensing measurements were carried-out on an intelligent gas sensing analysis system (CGS-1TP, Beijing Elite Tech Co., Ltd, China). The saturated target gas was introduced into the test chamber of 18 liters by a micro-injector using a rubber plug. Hence, the sensors resistance were collected and explored by the system in real time. The sensor temperature adjusted conductively with an accuracy of 1 °C by the analysis system presented an external temperature control (from room temperature to 500 °C) and the relative humidity in the system could be controlled by a dehumidifier. The sensing response (Sr) towards target gas was defined as R_g_/R_a_, where R_a_ and R_g_ are the resistance of the sensor in the presence of air and target gas, respectively. Herein, the gas-sensing measurements were carried out at a functioning temperature of 169 °C with optimized relative humidity of 45%.

## Electronic supplementary material

Supporting Information

## References

[1] Pauling L, Robinson AB, Teranishi R, Cary P (1971). Quantitative analysis of urine vapor and breath by gas-liquid partition chromatography. J. Proc. Natl. Acad. Sci. USA.

[2] Miekisch W, Schubert JK, Noeldge-Schomburg GF (2004). Diagnostic potential of breath analysis focus on volatile organic compounds. J. Clin. Chim. Acta.

[3] Guo D, Zhang D, Li N, Zhang L, Yang J (2010). A novel breath analysis system based on electronic olfaction. J. IEEE Trans. Biomed. Eng..

[4] Plaza M (2010). Screening for bioactive compounds from algae. J. pharm. biomed. Anal..

[5] Dummer JF (2010). Accurate, reproducible measurement of acetone concentration in breath using selected ion flow tube-mass spectrometry. J. Breath Res..

[6] Phillips M (1999). Volatile organic compounds in breath as markers of lung cancer: a cross-sectional study. J. The Lancet.

[7] Lei Z, Yang Y (2014). A Concise Colorimetric and Fluorimetric Probe for Sarin Related Threats Designed via the “Covalent-Assembly” Approach. J. Am. Chem. Soc..

[8] Xu Q (2013). Polydiacetylene-based colorimetric and fluorescent chemosensors for the detection of carbon dioxide. J. Am. Chem. Soc..

[9] Das S, Jayaraman V (2014). SnO_2_: A comprehensive review on structures and gas sensors. J. Prog. Mater. Sci..

[10] Yang H, Tao Q, Zhang X, Tang A, Ouyang J (2008). Solid-state synthesis and electrochemical property of SnO_2_/NiO nanomaterials. J. Alloys and Comp..

[11] Dharmaraj N (2006). Synthesis of nickel oxide nanoparticles using nickel acetate and poly (vinyl acetate) precursor. J. Mater. Sci. and Eng. B.

[12] Shi C, Wang G, Zhao N, Du X, Li J (2008). NiO nanotubes assembled in pores of porous anodic alumina and their optical absorption properties. J. Chem. Phys. Lett..

[13] Li L, He S, Liu M, Zhang C, Chen W (2015). Three-dimensional mesoporous graphene aerogel-supported SnO_2_ nanocrystals for high-performance NO_2_ gas sensing at low temperature. J. Anal. Chem..

[14] Kim HJ (2013). Ultra-selective and sensitive detection of xylene and toluene for monitoring indoor air pollution using Cr-doped NiO hierarchical nanostructures. J. Nanoscale.

[15] Wang C (2015). Ultrasensitive and low detection limit of acetone gas sensor based on W-doped NiO hierarchical nanostructure. J. Sens. Actuators, B: Chem..

[16] Sun X (2015). Enhanced gas-sensing performance of Fe-doped ordered mesoporous NiO with long-range periodicity. J. Phys. Chem. C.

[17] Gao G (2011). Synthesis of single-crystalline α-Fe_2_O_3_ nanobelts via a facile PEG-200 assisted solution route. J. Cryst. Eng. Comm..

[18] Kavitha T, Yuvaraj H (2011). A Facile Approach to the Synthesis of High-Quality NiO Nanorods: Electrochemical and Antibacterial Properties. J. Mater. Chem..

[19] Chen JS, Zhun T, Yang XH, Yang HG, Lou XW (2010). Top-Down Fabrication of α-Fe_2_O_3_ Single-Crystal Nanodiscs and Microparticles with Tunable Porosity for Largely Improved Lithium Storage Properties. J. Am. Chem. Soc..

[20] Kim HJ, Choi KI, Kim KM, Na CW, Lee JH (2012). Highly Sensitive C_2_H_5_OH Sensors Using Fe-Doped NiO Hollow Spheres. J. Sens. Actuators, B: Chem..

[21] Flynn CJ (2014). Hierarchically-structured NiO nanoplatelets as mesoscale p-type photocathodes for dye-sensitized solar cells. J. Phys. Chem. C.

[22] Niu HH (2013). Dye-sensitized solar cells based on flower-shaped α-Fe_2_O_3_ as a photoanode and reduced graphene oxide-polyaniline composite as a counter electrode. J. RSC Adv..

[23] Bai GM, Dai HX, Deng JG, Liu YX, Ji KM (2012). Porous NiO nanoflowers and nanourchins: highly active catalyst for toluene combustion. J. Catal. Commun..

[24] Ouyang JJ, Pei J, Kuang Q, Xie ZX, Zheng LS (2014). Supersaturation-controlled shape evolution of α-Fe_2_O_3_ nanocrystals and their facet- dependent catalytic and sensing properties. J. ACS Appl. Mater. Inter..

[25] Liu B (2011). Synthesis and Enhanced Gas-Sensing Properties of Ultralong NiO Nanowires Assembled with NiO Nanocrystals. J. Sens. Actuators, B: Chem..

[26] Sun P (2012). Gas sensing with hollow α-Fe_2_O_3_ urchin-like spheres prepared via template-free hydrothermal synthesis. J. Cryst. Eng. Comm..

[27] Ma JM (2012). NiONanomaterials: Controlled Fabrication, Formation Mechanism and the Application in Lithium-Ion Battery. . J. Cryst. Eng. Comm..

[28] Zhang P, Guo ZP, Liu HK (2010). Submicron-Sized Cube-Like α-Fe_2_O_3_ Agglomerates as an Anode Material for Li-Ion Batteries. J. Electrochim. Acta.

[29] Cui YF (2011). Lotus-Root-Like NiO Nanosheets and Flower-Like NiO Microspheres: Synthesis and Magnetic Properties. J. Cryst. Eng. Comm..

[30] Zhu LP, Xiao HM, Liu XM, Fu SY (2006). Template-Free Synthesis and Characterization of Novel 3D Urchin-Like α-Fe_2_O_3_ Superstructures. J. Mater. Chem..

[31] Kim SI, Lee JS, Ahn HJ, Song HK, Jang JH (2013). Facile Route to an Efficient NiO Supercapacitor with a Three-Dimensional Nano-network Morphology. J. ACS Appl. Mater. Inter..

[32] Chaudhari S, Bhattacharjya D, Yu JS (2013). 1-Dimensional Porous α-Fe_2_O_3_ Nanorods as High Performance Electrode Material for Supercapacitors. J. RSC Adv..

[33] Jain K, Pant RP, Lakshmikumar ST (2006). Effect of Ni doping on thick film SnO_2_ gas sensor. J. Sens. Actuators, B: Chem..

[34] Li L, Zhang C, Chen W (2015). Fabrication of SnO_2_–SnO nanocomposites with p–n heterojunctions for the low-temperature sensing of NO_2_ gas. J. Nanoscale.

[35] Kim HR (2011). The role of NiO doping in reducing the impact of humidity on the performance of SnO_2_-based gas sensors: synthesis, strategies, and phenomenological and spectroscopic studies. J. Adv. Funct. Mater..

[36] Deng, R. *et al*. X-ray photoelectron spectroscopy measurement of n-ZnO/p-NiO heterostructure valence-band offset. *J. Appl. Phys*. **94**, 022108-1-3 (2009).

[37] Yu M, Wu R, Chavali M (2011). Effect of ‘Pt’ loading in ZnO–CuO hetero-junction material sensing carbon monoxide at room temperature. J. Sens. Actuators, B: Chem..

[38] Barreca D (2011). Novel synthesis and gas sensing performance of CuO–TiO_2_ nanocomposites functionalized with Au nanoparticles. J. Phys. Chem. C.

[39] Chen YC (2012). Facile Procedure to Synthesize Highly Crystalline Ag/NiO Nanocomposite Microspheres and Their Photocatalytic Activity. J. Mater. Sci.: Mater. Electron..

[40] Sun P (2011). Synthesis and Gas Sensing Properties of Bundle-Like α-Fe_2_O_3_ Nanorods. J. Sens. Actuators, B: Chem..

[41] Sugiyama I (2013). Ferromagnetic Dislocations in Antiferromagnetic NiO. J. Nat. Nanotechnol..

[42] Wu P, Sun JH, Huang YY, Gu GF, Tong DG (2012). Solution Plasma Synthesized Nickel Oxide Nanoflowers: An Effective NO_2_ Sensor. J. Mater. Lett..

[43] Bai GM (2013). The Microemulsion Preparation and High Catalytic Performance of Mesoporous NiO Nanorods and Nanocubes for TolueneCombustion. J. Chem. Eng..

[44] Needham SA, Wang GX, Liu HK (2006). Synthesis of NiO Nanotubes for Use as Negative Electrodes in Lithium Ion Batteries. J. Power Sources.

[45] Liu S (2012). Synthesis of Fe-doped NiO nanofibers using electrospinning method and their ferromagnetic properties. J. Magnet. Mater..

[46] Hidalgo P, Castro RHR, Coelho ACV, Gouvea D (2005). Surface segregation and consequent SO_2_ sensor response in SnO_2_–NiO. J. Chem. Mater..

[47] Choi JK (2010). Design of selective gas sensors using electrospun Pd-doped SnO_2_ hollow nanofibers. J. Sens. Actuators, B: Chem..

[48] Grosvenor AP, Kobe BA, Biesinger MC, Mclntyre NS (2004). Investigation of multiplet splitting of Fe 2p XPS spectra and bonding in iron compounds. J. Surf. Inter. Anal..

[49] Zhao B (2009). Synthesis of flower-like NiO and effects of morphology on its catalytic properties. J. Phys. Chem. C.

[50] Pan JH (2013). Scalable synthesis of urchin-and flowerlike hierarchical NiO microspheres and their electrochemical property for lithium storage. . J. ACS Appl. Mater. Inter..

[51] Li W (2016). MOF-derived hierarchical hollow ZnO nanocages with enhanced low-concentration VOCs gas-sensing performance. J. Sens. Actuators, B: Chem..

[52] Heiland G, Kohl D (1988). Physical and chemical aspects of oxidic semiconductor gas sensors. J. Chem. Sen. Tech..

[53] Bai Z, Xie C, Hu M, Zhang S, Zeng D (2008). Effect of humidity on the gas sensing property of the tetrapod-shaped ZnO nanopowder sensor. J. Mater. Sci. and Eng. B.

[54] Liu C (2016). Facile synthesis and gas sensing properties of the flower-like NiO-decorated ZnO microstructures. J. Sens. Actuators, B: Chem..

[55] Liu Y (2014). An environment benign method for the synthesis of p-NiO/n-ZnO heterostructure with excellent performance for gas sensing and photo-catalysis. J. Sens. Actuators, B: Chem..

[56] Zhang B (2017). Enhanced gas sensing properties to acetone vapor achieved by α-Fe_2_O_3_ particles ameliorated with reduced graphene oxide sheets. J. Sens. Actuators, B: Chem..

[57] Kim HJ, Lee JH (2014). Highly sensitive and selective gas sensors using p-type oxide semiconductors: Overview. J. Sens. Actuators, B: Chem..

[58] Ju DX (2015). NearRoom Temperature, Fast-Response, and Highly Sensitive Triethylamine Sensor Assembled with Au-Loaded ZnO/SnO2 Core–Shell Nanorods on Flat Alumina Substrates. J. ACS Appl. Mater. Inter..

[59] Li L, Liu M, He S, Chen W (2014). Freestanding 3D mesoporous Co_3_O_4_@ carbon foam nanostructures for ethanol gas sensing. J. Anal. chem..

[60] Wang YS (2014). Brookite TiO_2_ decorated α-Fe_2_O_3_ nano-heterostructures with rod morphologies for gas sensor application. J. Mater. Chem. A.

[61] Itoh E, Shirotori T (2012). Relationship between work function of hole collection electrode and temperature dependence of open-circuit voltage in multilayered organic solar cells. Jpn. J. Appl. Phys..

[62] Kawade D, Chichibu SF, Sugiyama M (2014). Experimental determination of band offsets of NiO-based thin film heterojunctions. J. Appl. Phys..

[63] Wu JQ, Deng SZ, Xu NS, Chen J (2014). Field emission from α-Fe_2_O_3_ nanoflakes: effect of vacuum pressure, gas adsorption and *in-situ* thermal treatment. J. Appl. Surf. Sci..

[64] Kennedy JH, Frese KW (1978). Flatband potentials and donor densities of polycrystalline α -Fe_2_O_3_ determined from mott-schottky plots. J. Electrochem. Soc..

[65] Xu Y, Schoonen MA (2000). The absolute energy positions of conduction and valence bands of selected semiconducting minerals. J. Am. Miner..

[66] Alenezi MR (2013). Role of the exposed polar facets in the performance of thermally and UV activated ZnO nanostructured gas sensors. J. Phys. Chem. C.

[67] Wen Z (2015). Rhombus-shaped Co_3_O_4_ nanorods arrays for high-performance gas sensor. J. Sens. Actuators, B: Chem..

