# Supplementary material for: A two-step synthesis of nanosheet-covered fibers based on α-Fe2O3/NiO composites towards enhanced acetone sensing
Source: Sci Rep. 2018 Jan 26;8:1705. doi: 10.1038/s41598-018-20103-y (PMC5786040; doi:10.1038/s41598-018-20103-y)
Supplement: Supplementary file 1 — Supporting Information [file 41598_2018_20103_MOESM1_ESM.pdf]

Supporting Information

A two-step synthesis of nanosheet-covered fibers based on α-Fe_2O_3/NiO composites towards enhanced acetone sensing

Mahmood ul Haq[1], *Zhen Wen*[2], *Ziyue Zhang*[1], *Shahid Khan*[1], *Zirui Lou*[1], *Zhizhen Ye*[1], *Liping Zhu*[1,]*,

[1]State Key Laboratory of Silicon Materials, School of Materials Science and Engineering, Cyrus Tang Center for Sensor Materials and Applications, Zhejiang University, Hangzhou, 310027, China.

[2]Institute of Functional Nano and Soft Materials (FUNSOM), Jiangsu Key Laboratory for Carbon-Based Functional Materials and Devices, and Collaborative Innovation Center of Suzhou Nano Science and Technology, Soochow University, Suzhou 215123, China.

* Corresponding author: Liping Zhu.

Tel.: +86 571 8795 1958.

E-mail address: zlp1@zju.edu.cn

Table of Content

Figure S1. (a, b) SEM images of composites S-1 based on *α*-Fe_2O_3/NiO nanosheet-covered fibers **(c, d)** SEM images of composites S-3 based on *α*-Fe_2O_3/NiO nanosheet-covered fibers.

Figure S2. Energy dispersive x-ray spectroscopy, (EDX's) elemental images **(a)** Pure NiO nanofibers and **(b)** Composites S-1, S-2 and S-3 based on α-Fe_2O_3/NiO nanosheet-covered fibers.

Figure S3. The XPS survey spectra of bare NiO nanofibers and composites S-2 based on *α*-Fe_2O_3/NiO nanosheet-covered fibers

Figure S4. (a) Response times and **(b)** Recovery times of NiO nanofibers and composites S-1, S-2 and S-3 based on *α*-Fe_2O_3/NiO nanosheet-covered fibers to 100 ppm acetone at 169 °C.

Figure S5. UV-visible spectrum and bandgap energy; **(a)** *α*-Fe_2O_3 nanosheets and **(b)** NiO nanofibers.

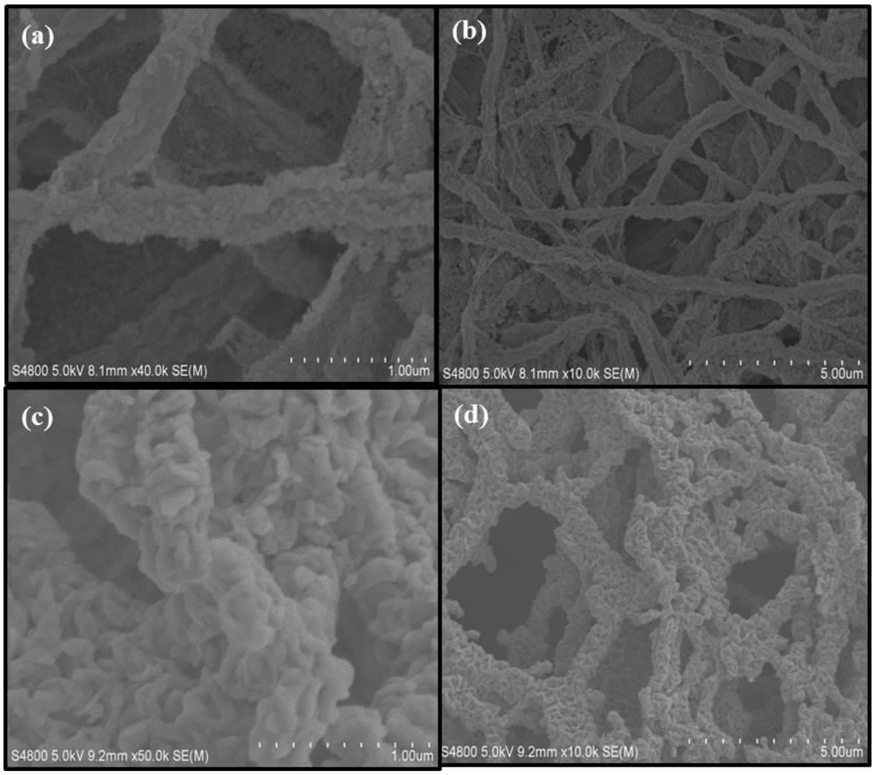

Figure S1. (a, b) SEM images of composites S-1 based on α-Fe_2O_3/NiO nanosheet-covered fibers **(c, d)** SEM images of composites S-3 based on α-Fe_2O_3/NiO nanosheet-covered fibers.

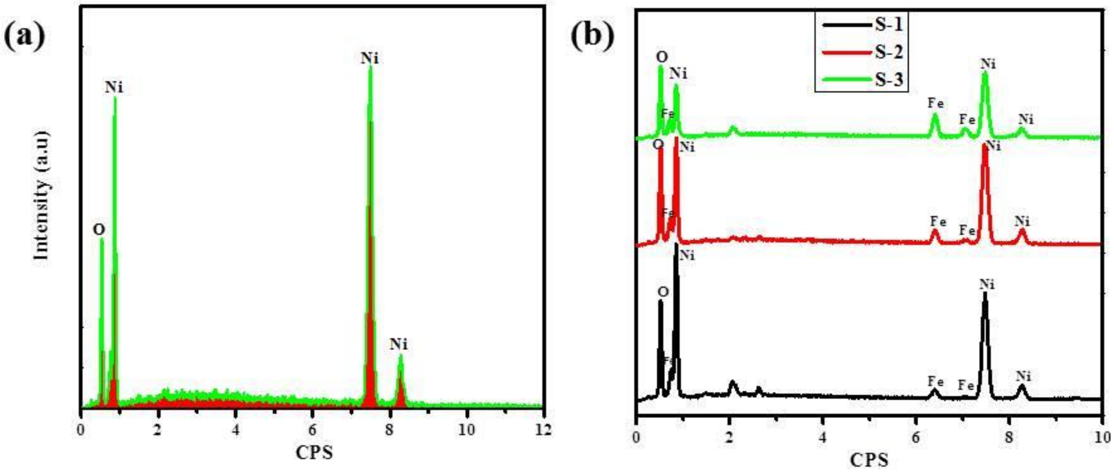

Figure S2. Energy dispersive x-ray spectroscopy, (EDX's) elemental images **(a)** Pure NiO nanofibers and **(b)** Composites S-1, S-2 and S-3 based on α-Fe_2O_3/NiO nanosheet-covered fibers.

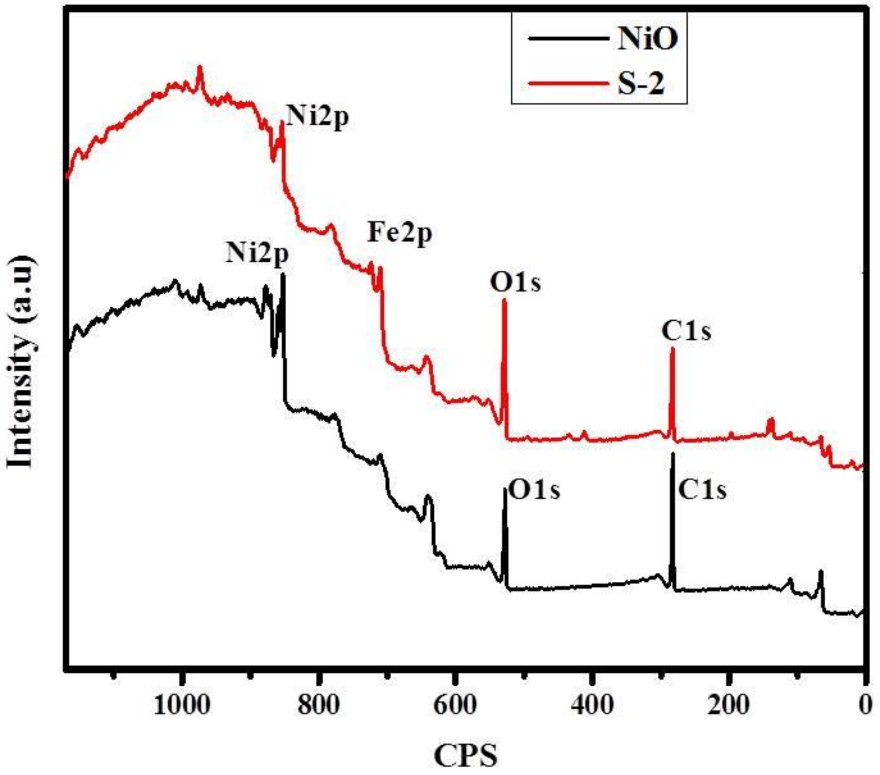

Figure S3. The XPS survey spectra of bare NiO nanofibers and composites S-2 based on α-Fe_2O_3/NiO nanosheet-covered fibers.

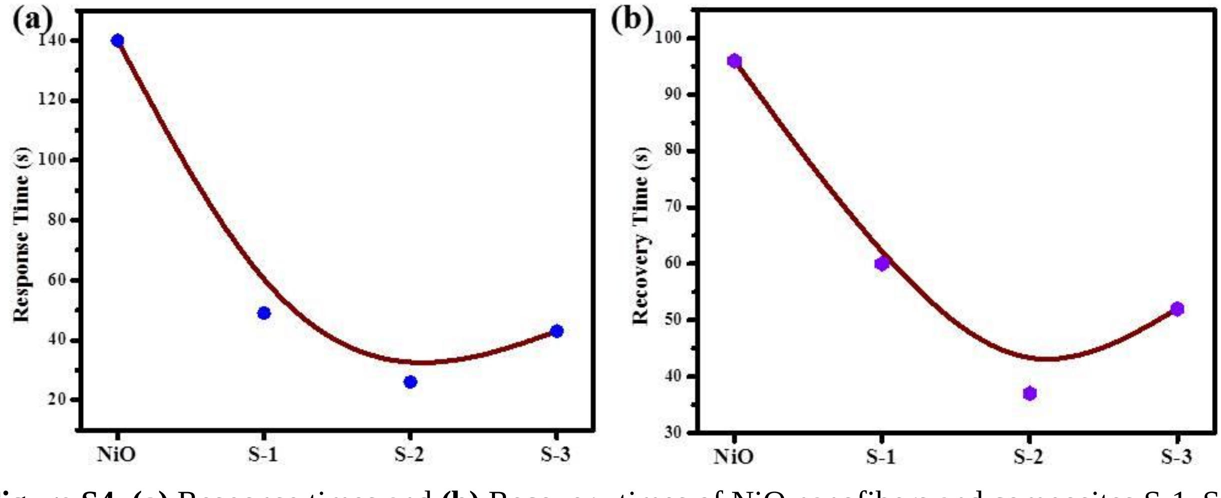

Figure S4. (a) Response times and **(b)** Recovery times of NiO nanofibers and composites S-1, S-2 and S-3 based on α-Fe_2O_3/NiO nanosheet-covered fibers to 100 ppm acetone at 169 °C.

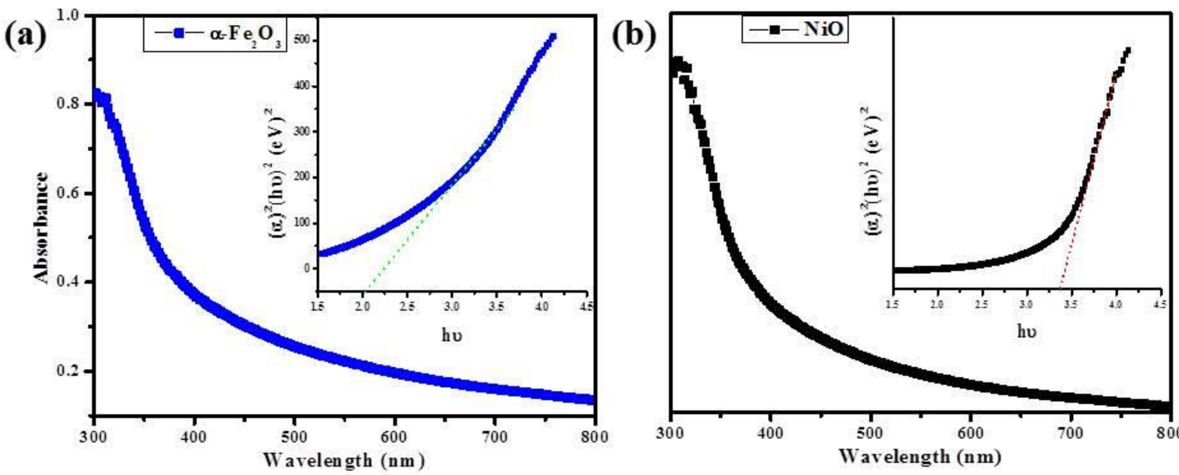

Figure S5.UV-visible spectrum and bandgap energy; **(a)** α-Fe_2O_3 nanosheets and **(b)** NiO nanofibers.
